# Scar Revision: Analysis of Post-Scar Revision Satisfaction and Parameters Decisive for Patients’ Satisfaction

**Published:** 2017-01

**Authors:** Patil Salil, Boro Sumanjit, Prajapati Chetan, Suri P Manav, Sachde P Jayesh, MF Shaikh

**Affiliations:** Department of Burns and Plastic Surgery, BJ Medical College and Civil Hospital Ahmedabad, Gujarat, India

**Keywords:** Scar revision, Patient satisfaction, India

**DEAR EDITOR**

Studies of quality of life measures in scar patients reveal significant interference with physical comfort as well as in social and professional life. The lay population often refers to a scar as an unsightly healed wound. Conversely, surgeons tend to think of surgical scars as an expected outcome of a violation of the dermis. Before initiating treatment, the physician must take the time to understand and diagnose each element. The extent of scar must be considered along with the patient’s intent in getting treatment.^1^

This is a prospective study of 50 cases of scar revision performed in our department from 2011- 2014. Mature scars more than 2mm in diameter broad were included in the study. Patients were analysed in terms of patient satisfaction with the post-operative outcome. Patients were followed up for a minimum period of 6 months. Exclusion criteria were (i) pregnant patients, (ii) paediatric patients and (iii) patients with a follow up period of <6 months. Patients were analysed regarding their satisfaction with their post-operative outcome in the form of scar related parameters and psychological parameters. Post-operative patient satisfaction was assessed using pre and post procedure Vancouver Scar Scale scores.^2^^,^^3^

The largest number of cases were seen in the age group 16-30 years (78%), of which 21 patients (53.8%) were satisfied with the outcome of their corrective procedure. The preponderance in this age group could be due to the fact that young adults are highly conscious regarding their appearance (Table 1). Among 24 female patients, 66.7% appeared to be more satisfied with the outcomes of corrective procedures as compared to 26 male patients (46.1%). The most common site for scar revision was cheek (32%) followed by the forehead (26%). Patients who underwent correctional procedures for cheek scars showed maximum satisfaction post-operatively (Table 2). 

**Table 1:** Satisfaction rate of patients in different age groups

**Age Group (Years)**	**Age distribution**	**Percentage **	**Satisfied **	**Not- satisfied**	**Same **
11 to 15	7	14	5	1	1
16 to 30	39	78	21	5	13
31 to 40	3	6	2	-	1
> 40	1	2	-	-	1

**Table 2:** Satisfaction rate of patients based on scar area

**Scar area**	**No. of patients**	**Percentage**	**Satisfied **	**Not-satisfied**	**Same **
Forehead	13	26	6	1	6
Eye	3	6	1	1	1
Cheek	16	32	10	2	4
Chin	6	12	4	1	1
Neck	3	6	1	0	2
Abdomen	1	2	1	0	0
Arm	3	6	2	0	1
Nose	4	8	2	1	1
Ear	1	2	1	0	0

The incidence of post-operative satisfaction was slightly higher among 25 unmarried enrolled patients (60%) compared to 25 studied married patients (52%). No significant difference in post-operative satisfaction could be found in patients with decreased (n=10) or unchanged scar visibility (n=10) following operative procedure. Scars which were converted to lie parallel to Relaxed Skin Tension Lines (RSTL) were seen to have the maximum satisfaction with post-operative results (83%) compared to other patients (72%). However the small number of patients in the category (5 and 8 patients) precludes any statistically significant difference to be obtained. Eighteen patients noticed gain of function of the affected part following scar revision (n=38).

All patients with gain of function in the affected part showed satisfaction with their post-operative outcome. The highest incidence of post-operative satisfaction correlates with anxiety relief following correctional procedure (84% of 13 enrolled patients). The highest post-operative satisfaction was seen to correlate with alleviation of depression following correctional procedures (89% of 19 studied patients). No significant difference was seen in post-operative satisfaction rates in patients with either increased or high self-esteem following surgical procedure. However patients with low self-esteem demonstrated lower satisfaction rates compared to their counterparts (40% vs. 56% and 60%). The highest percentage of post-operative satisfaction was seen in patients with improved social acceptance following correctional procedure (73%) when compared to others (50% and 30%). 

Patients with change of 4 or more between pre and post operative Vancouver Scar Scale scores were satisfied with the outcome of the revisional procedures while those with scores less than 4 were either dissatisfied or neither satisfied nor dissatisfied with the outcome. Post-operative satisfaction in an individual is multifactorial. Females appear to be marginally more satisfied as compared to males with their post operative outcomes. Patients who underwent correctional procedures for cheek scars showed maximum satisfaction post operatively probably because the cheek is an exposed area and defects if an are promptly visible. Even a small improvement in a scar in such a cosmetically important area can bring great satisfaction to the patient.

Patients in whom scars came to lie parallel to or within RSTL’s post operatively, as compared to pre-operative status in which they did not, showed the maximum level of satisfaction with their corrective procedures.^4^^,^^5^ Following scar revisional procedures, marked reduction in anxiety and depression as compared to pre-operative status was noticed. Also, patients with low self esteem tended to be unsatisfied with their post operative outcome. Increased social acceptance, either real or apparent, could be a major factor leading to this conclusion. 

## CONFLICT OF INTEREST

The authors declare no conflict of interest.
